# Surgical Strategies for Tuberculous Spondylodiscitis With Severe Osteoporosis: A Case Illustrating the Challenges of Delayed Intervention

**DOI:** 10.7759/cureus.49021

**Published:** 2023-11-18

**Authors:** Diogo Luz, Carla Sousa, Melissa Silva, Pedro Pais, Vitor C Ferreira

**Affiliations:** 1 Neurosurgery, Hospital Garcia de Orta, Almada, PRT

**Keywords:** lumbopelvic instability, anterior lumbar corpectomy, case report, surgical intervention, severe osteoporosis, pott's spine, tuberculous spondylodiscitis

## Abstract

Tuberculous spondylodiscitis (Pott's spine) is a complex extrapulmonary manifestation of tuberculosis (TB) that poses significant medical challenges, characterized by vertebral destruction affecting approximately 2% of all TB cases. The management of this condition involves a multidisciplinary approach, with surgical intervention indicated for specific cases, including those with neurological complications, spinal instability, and kyphosis. We report a case of a 49-year-old female with a confirmed diagnosis of tuberculous spondylodiscitis who had undergone eight months of tuberculostatic therapy. She was referred for neurosurgical consultation due to uncontrollable axial pain, despite favorable clinical and imaging responses, which had rendered her immobile for six months. Imaging revealed a complete collapse of the L5 vertebral body, and a complementary dual x-ray absorptiometry (DEXA) scan demonstrated severe osteoporosis. A two-stage surgical approach was chosen to address her condition, involving corpectomy through an anterior approach, followed by lumbopelvic stabilization. Postoperative recovery was uneventful, with progressive improvement in pain and mobility. This case highlights the challenges of managing tuberculous spondylodiscitis and underscores the significance of early detection to prevent complications like severe osteoporosis. In this case, delayed referral for surgery following an extended period of immobility added complexity to an already difficult case. The severe osteoporosis, with a t-score of -5.7, had a substantial impact on surgical planning, leading to a more robust approach to arthrodesis with substantial lumbopelvic instrumentation in order to mitigate the risks associated with implant failure. This case shows that timely intervention and a comprehensive multidisciplinary approach are essential for the effective management of tuberculous spondylodiscitis, especially in cases complicated by severe osteoporosis.

## Introduction

Spinal tuberculosis (TB), often referred to as Pott's spine, is a severe manifestation of extrapulmonary TB. The pioneering description of tuberculous spondylitis by Sir Percivall Pott in 1779 marked the inception of understanding this intricate pathology [1]. Characterized by vertebral destruction, spinal TB comprises 2% of all TB cases, contributing to 15% of extrapulmonary and 50% of skeletal TB instances [1-3].

The management of spinal TB encompasses a multidisciplinary approach, wherein antitubercular drug therapy forms the cornerstone, while surgical intervention is reserved for specific indications in cases with cold abscesses, neurological complications, spinal instability, kyphosis, and treatment-resistant situations [1]. Surgical techniques aim at debridement, neural decompression, correction of deformity, and spinal stabilization [1,2].

The use of advanced imaging techniques such as computer tomography (CT) and magnetic resonance imaging (MRI) has enabled the identification of tuberculous spinal infections in the pre-destructive or early destructive phase, thus allowing conservative treatment [4]. However, conservative approaches may fail due to unresponsiveness or noncompliance with drug therapy, leading to complications such as pathologic kyphosis and neurological deficits [4]. Kyphotic deformity remains a concern, necessitating the exploration of surgical interventions for select patients [4]. Aggressive surgical strategies in the management of spinal TB play an essential role in stabilizing the affected vertebral segment, promoting recovery, and mitigating long-term consequences [4].

This case report illustrates the complexities of managing tuberculous spondylodiscitis, highlighting the challenges posed by severe osteoporosis caused by delayed treatment and neurological involvement. The case underscores the significance of surgical interventions in managing this intricate pathology.

## Case presentation

A 49-year-old obese female with a confirmed diagnosis of L5 tuberculous spondylitis underwent eight months of tuberculostatic therapy. The diagnosis of *Mycobacterium tuberculosis *had been previously established through PCR-DNA of a CT-guided biopsy sample. She had a history of smoking and had experienced thromboembolism three years ago. She was then referred for neurosurgical consultation for uncontrollable axial pain, rendering her immobile and bedridden for the past six months.

Imaging findings

**Figure 1:**
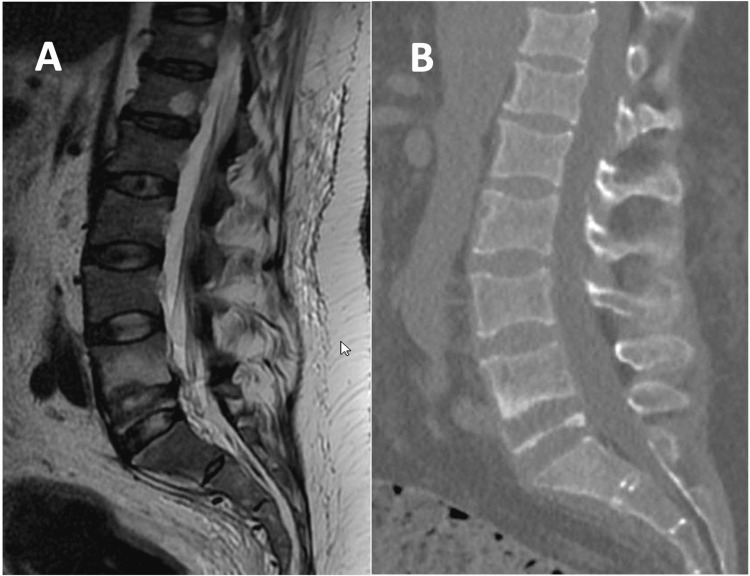
Imaging findings Lumbar spine MRI (A) and CT scan (B) showing complete collapse of the vertebral body of L5. CT, computer tomography; MRI, magnetic resonance imaging

CT and MRI assessments indicated successful treatment of TB, with no abscesses noted, consistent with prior assessments (Figure 1). However, a concerning progression was observed as evidenced by the complete collapse of the L5 vertebral body. Subsequently, recognizing the prolonged immobility period and apparent sarcopenia, bone densitometry was ordered. The results revealed severe osteoporosis, indicated by a femoral t-score of -5.7.

Surgical approach

**Figure 2:**
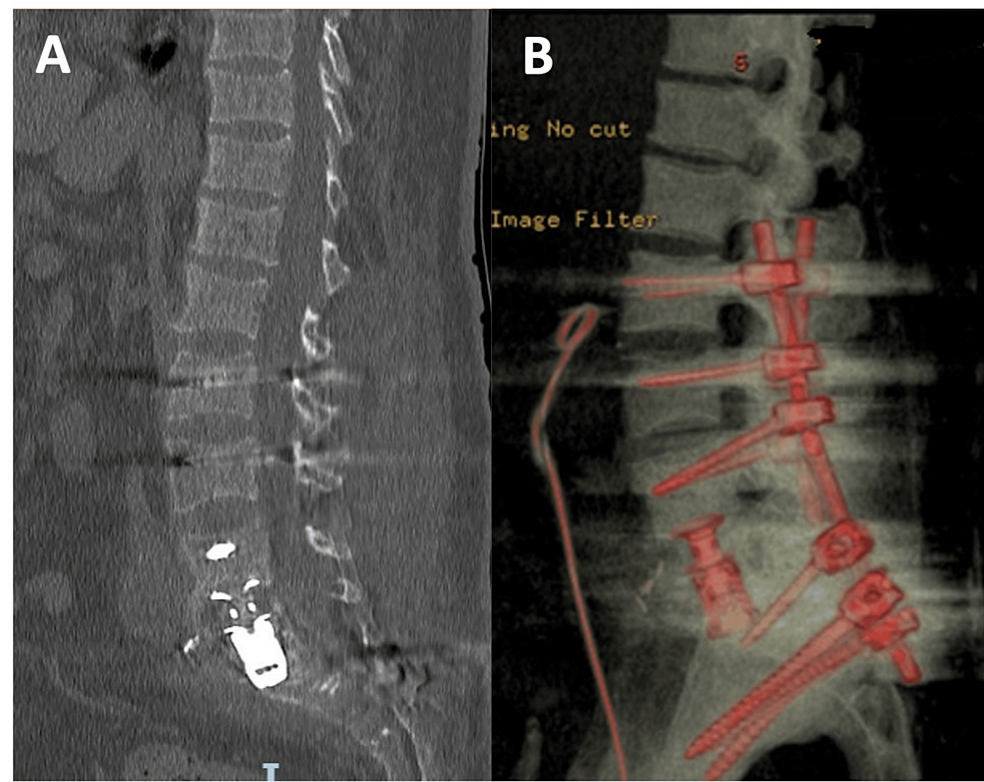
Postoperative imaging First-day postoperative sagittal midline CT scan (A) and 3D reconstruction (B) of the construct. The construct is composed of bilateral pedicular screws L2-L4, with pelvic instrumentation with iliac screws and S2AI screws bilaterally. S2AI, S2 Alar Iliac; CT, computer tomography

A two-stage surgical intervention of an L2-pelvic arthrodesis was chosen to address the patient's condition. In the first stage, a corpectomy of the L5 vertebra through an anterior approach was performed, along with the placement of an expandable cylinder. In the second stage, lumbopelvic stabilization was achieved using transpedicular lumbar instrumentation and iliac wing screw placement. Figure 2 shows the postoperative CT scan and a 3D image of the construct.

Postoperative course

**Figure 3:**
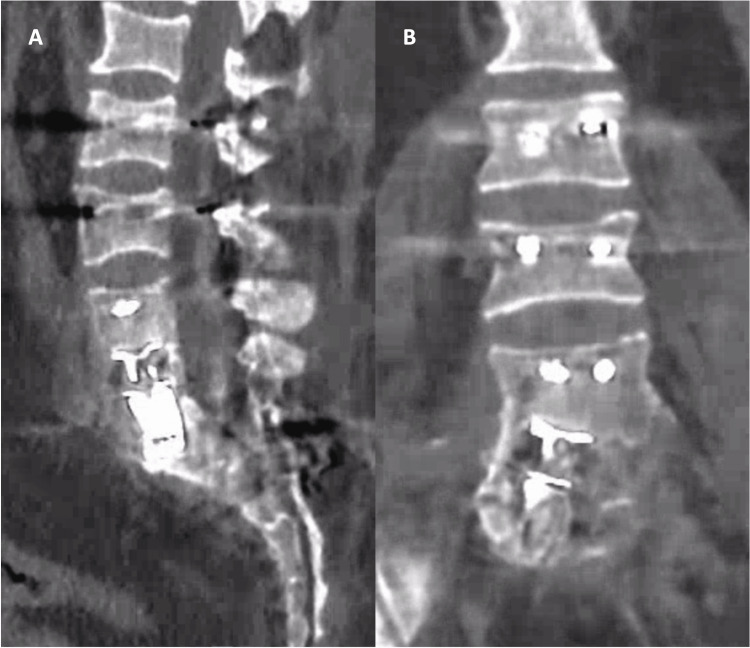
A 24-month follow-up CT scan Sagittal (A) and coronal (B) views of 24-month follow-up CT scan. The scan illustrates bony fusion achieved through the expandable cage. Notably, a mild cage subsidence through the superior endplate of S1 is observed; however, it did not appear to impact the overall positive outcome in this case. CT, computer tomography

Postoperative recovery was uneventful, with no surgical complications observed. The patient commenced motor rehabilitation, experiencing progressive improvement in pain and mobility. A six-month follow-up revealed a notable decrease in axial pain, with the standing lumbar pain Visual Analog Scale (VAS) improving from nine to five. Furthermore, there was enhanced overall muscle strength, reaching a grade of 4+ in the Medical Research Council (MRC) scale for leg strength, bilaterally. Importantly, a 24-month CT scan documented successful fusion (Figure 3), confirming the positive outcomes of the surgical intervention.

## Discussion

The presented case offers valuable insights into the intricate nature of managing tuberculous spondylodiscitis and underscores the multifaceted challenges it presents. In this context, the case emphasizes the significance of early detection to prevent complications such as severe osteoporosis, which can significantly impact treatment strategies and outcomes. 

The case underscores a significant challenge of late referral to neurosurgery following an extended period of TB treatment. Despite the successful management of TB, the patient's persistent axial pain led to prolonged immobilization, likely contributing to the development of severe osteoporosis, as indicated by a t-score of -5.7 on bone densitometry - a factor well-documented to have a substantial impact on surgical outcomes [5-7]. Osteoporosis, characterized by reduced bone mineral density, introduces an elevated risk of implant failure following spinal procedures, particularly in regions like the lumbosacral junction, which demands substantial mechanical stability [5]. This scenario highlights the paramount importance of early referral for patients with vertebral osteomyelitis. In this case, early intervention could have potentially averted the high surgical risk associated with severe osteoporosis, offering a more favorable prognosis.

Given the patient's osteoporotic condition and the associated risk factors, a robust approach to arthrodesis with substantial instrumentation became imperative. As documented in the literature, high lordotic angles, as observed in the lumbosacral junction, along with reduced bone mineral density, substantially increase the risk of implant failure after procedures like a vertebral body corpectomy [5]. To mitigate these risks and improve the patient's chances of a successful outcome, a comprehensive surgical plan was devised. Drawing from the approach outlined by Fukuta et al., a two-stage surgical strategy was chosen [8]. This strategy involved the initial phase of anterior debridement and corpectomy at the L5 level, incorporating the placement of an expanding cylinder to address the vertebral body collapse. Subsequently, the second stage encompassed posterior lumbopelvic instrumentation. This combination of surgical techniques aimed to provide structural support and stability.

The outcome was in line with the documented cases ﻿with an immediate pain improvement after the first surgery, with continued decreases in the follow-up period [8].

## Conclusions

In conclusion, this case underscores the intricate challenges of managing tuberculous spondylodiscitis, particularly in cases complicated by severe osteoporosis and delayed referral to neurosurgery. The unique aspects of our patient, combined with the utilization of a two-stage surgical strategy involving anterior debridement and corpectomy at the L5 level, along with posterior lumbopelvic instrumentation, distinguish this report from existing literature. Notably, the fusion outcomes documented in our case further contribute to the limited pool of published cases with similar characteristics. With early detection, meticulous surgical planning, and a multidisciplinary approach, clinicians can navigate the complexities of tuberculous spondylodiscitis more effectively, ultimately improving patient outcomes. This report fills a gap in the literature by presenting an underpublished type of patient and delineating surgical strategies tailored to their unique challenges.
